# Intronic regulation of SARS-CoV-2 receptor (ACE2) expression mediated by immune signaling and oxidative stress pathways

**DOI:** 10.1016/j.isci.2022.104614

**Published:** 2022-06-15

**Authors:** Daniel Richard, Pushpanathan Muthuirulan, Jennifer Aguiar, Andrew C. Doxey, Arinjay Banerjee, Karen Mossman, Jeremy Hirota, Terence D. Capellini

**Affiliations:** 1 Department of Human Evolutionary Biology, Harvard University, Cambridge, MA 02138, USA; 2 Department of Biology, University of Waterloo, Waterloo, ON N2L3G1, Canada; 3 Vaccine and Infectious Disease Organization, University of Saskatchewan, Saskatoon, SK S7N 5E3, Canada; 4 Department of Veterinary Microbiology, Western College of Veterinary Medicine, University of Saskatchewan, Saskatoon, SK S7N5B4, Canada; 5 Department of Medicine, McMaster University, Hamilton, ON L8N 3Z5, Canada; 6 Division of Respiratory Medicine, Department of Medicine, University of British Columbia, Vancouver, BC V5Z 1M9, Canada; 7 Broad Institute of MIT and Harvard, Cambridge, 02142 MA, USA

**Keywords:** Biological sciences, Molecular biology, Immunology, Virology

## Abstract

The angiotensin-converting enzyme 2 (ACE2) protein is a key catalytic regulator of the renin-angiotensin system (RAS), involved in fluid homeostasis and blood pressure modulation. ACE2 also serves as a cell-surface receptor for some coronaviruses such as *SARS-CoV* and *SARS-CoV-2*. Improved characterization of *ACE2* regulation may help us understand the effects of pre-existing conditions on COVID-19 incidence, as well as pathogenic dysregulation following viral infection. Here, we perform bioinformatic analyses to hypothesize on *ACE2* gene regulation in two different physiological contexts, identifying putative regulatory elements of *ACE2* expression. We perform functional validation of our computational predictions via targeted CRISPR-Cas9 deletions of these elements *in vitro*, finding them responsive to immune signaling and oxidative-stress pathways. This contributes to our understanding of *ACE2* gene regulation at baseline and immune challenge. Our work supports pursuit of these putative mechanisms in our understanding of infection/disease caused by current, and future, SARS-related viruses such as *SARS-CoV-2*.

## Highlights

• Lung expression patterns suggest *ACE2* regulation by immune and oxidative signaling
• CRISPR deletion of intronic regulatory elements (REs) alters *ACE2* expression
• Effects of RE deletion are modified by immune stimulation and oxidative stress
• Propose two mechanisms for regulating *ACE2* at baseline and after immune challenge

## Graphical abstract

**Figure:**
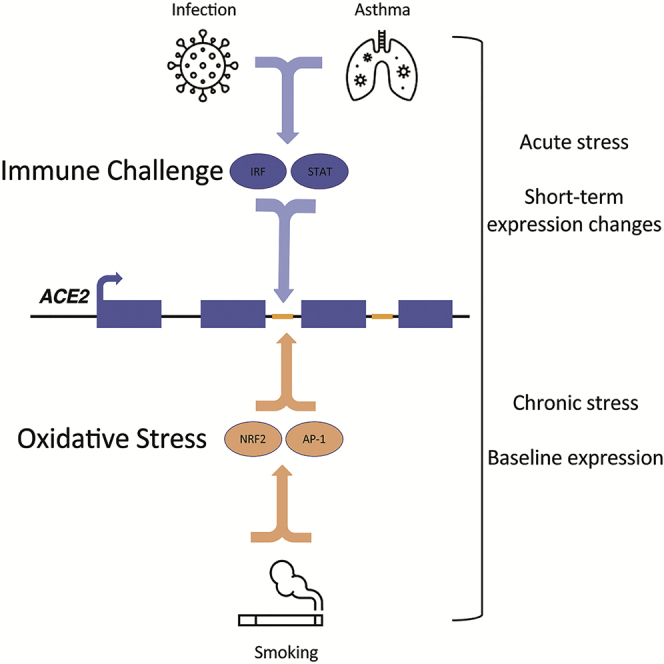

## Introduction

The angiotensin-converting enzyme 2 (ACE2) protein has been highly studied as a key catalytic regulator of the renin-angiotensin system (RAS), involved in fluid homeostasis and blood pressure modulation (Lavoie and Sigmund, 2003). ACE2 control on this system occurs both directly (i.e., by lowering levels of angiotensin II) and indirectly (i.e., via alternative cleavage products) inhibiting the self-damaging effects of RAS overactivation, including vasoconstriction, fibrosis, and excessive inflammation (Gheblawi et al., 2020). The RAS system functions across different organs (Lavoie and Sigmund, 2003), and similarly, ACE2 is expressed throughout the body (Aguiar et al., 2020; Hikmet et al., 2020) where it mediates its protective effects and impacts tissue function (Gheblawi et al., 2020). This activity has prompted the pursuit of ACE2 as a clinical target for protection and treatment against cardiovascular disease, diabetes mellitus, and acute lung damage (Gheblawi et al., 2020; Wang et al., 2020).

In addition to its important physiological role as a broadly expressed membrane-bound protein (Gheblawi et al., 2020), ACE2 serves as a cell-surface receptor for some viruses—most notably, coronaviruses such as *SARS-CoV* (Li et al., 2003) and *SARS-CoV-2* (Gheblawi et al., 2020; Wang et al., 2020; Zhou et al., 2020). Protein overexpression studies have demonstrated that ACE2 facilitates *SARS-CoV-2* infection (Zhou et al., 2020), while mice with engineered human ACE2 are susceptible to infection (Yinda et al., 2021), and it has been suggested that the distribution of ACE2 receptor expression across different tissues contributes to differential virus susceptibility (e.g., lung tissue and alveolar cells) (Zhang et al., 2020a). The tissue expression of ACE2 may also explain the wide-ranging symptoms of COVID-19 in patients (Clerkin et al., 2020), though alternative means of viral entry has been suggested (Aguiar et al., 2020). During infection, ACE2 proteins bound by *SARS-CoV-2* particles are endocytosed which, along with increased ADAM17 activity and upstream transcriptional changes, lead to a depletion of cell-surface ACE2 localization and reduced angiotensin catalytic activity (Clerkin et al., 2020; Wang et al., 2020). It has been suggested that reduced expression of *ACE2* may lead to an imbalance of the RAS system in patients with COVID-19, which may represent a major pathological outcome of viral infection (Gheblawi et al., 2020; Lanza et al., 2020). These findings have prompted intense interest in the use of recombinant ACE2 and other synthetic mimics as potential therapeutics (Hippisley-Cox et al., 2020; Monteil et al., 2021; Zhang et al., 2020b).

Given that levels of ACE2 expression may impact viral susceptibility (Aguiar et al., 2020; Devaux et al., 2020), and that subsequent changes to expression is a likely pathogenic mechanism of disease (Gheblawi et al., 2020; Lanza et al., 2020), an improved understanding of how *ACE2* expression is regulated at the genomic and transcriptional level may help us understand not only how the effects of pre-existing conditions (e.g., chronic obstructive pulmonary disease, (COPD)) may manifest with increased COVID-19 incidence but also the mechanisms that regulate ACE2 levels following viral infection (Bhalla et al., 2020; Ni et al., 2020a; Wang et al., 2022). In this study, we first perform bioinformatic analyses of several datasets to generate hypotheses about *ACE2* gene-regulatory mechanisms in the context of immune signaling and chronic oxidative stress. We next identify putative non-coding regulatory elements within the intronic regions of the *ACE2* gene as potential determinants of *ACE2* expression activity. We then perform functional validation of our computational predictions via targeted deletion of the identified *ACE2 cis*-regulatory elements in the context of immunological stimulation and oxidative stress conditions. Our results demonstrate the presence of intronic *ACE2* regulatory elements responsive to both immune signaling and oxidative-stress pathways, contributing to our understanding of how expression of this gene may be modulated at both baseline and during immune challenge. Furthermore, our work supports the further pursuit of these putative mechanisms in our understanding, prevention, and treatment of infection and disease caused by ACE2-utilizing viruses such as *SARS-CoV*, *SARS-CoV-2*, and future emerging SARS-related viruses (Wells et al., 2021).

## Results

### Upregulation of *ACE2* gene expression in healthy individuals is associated with immune signaling and viral infection

**Figure 1:**
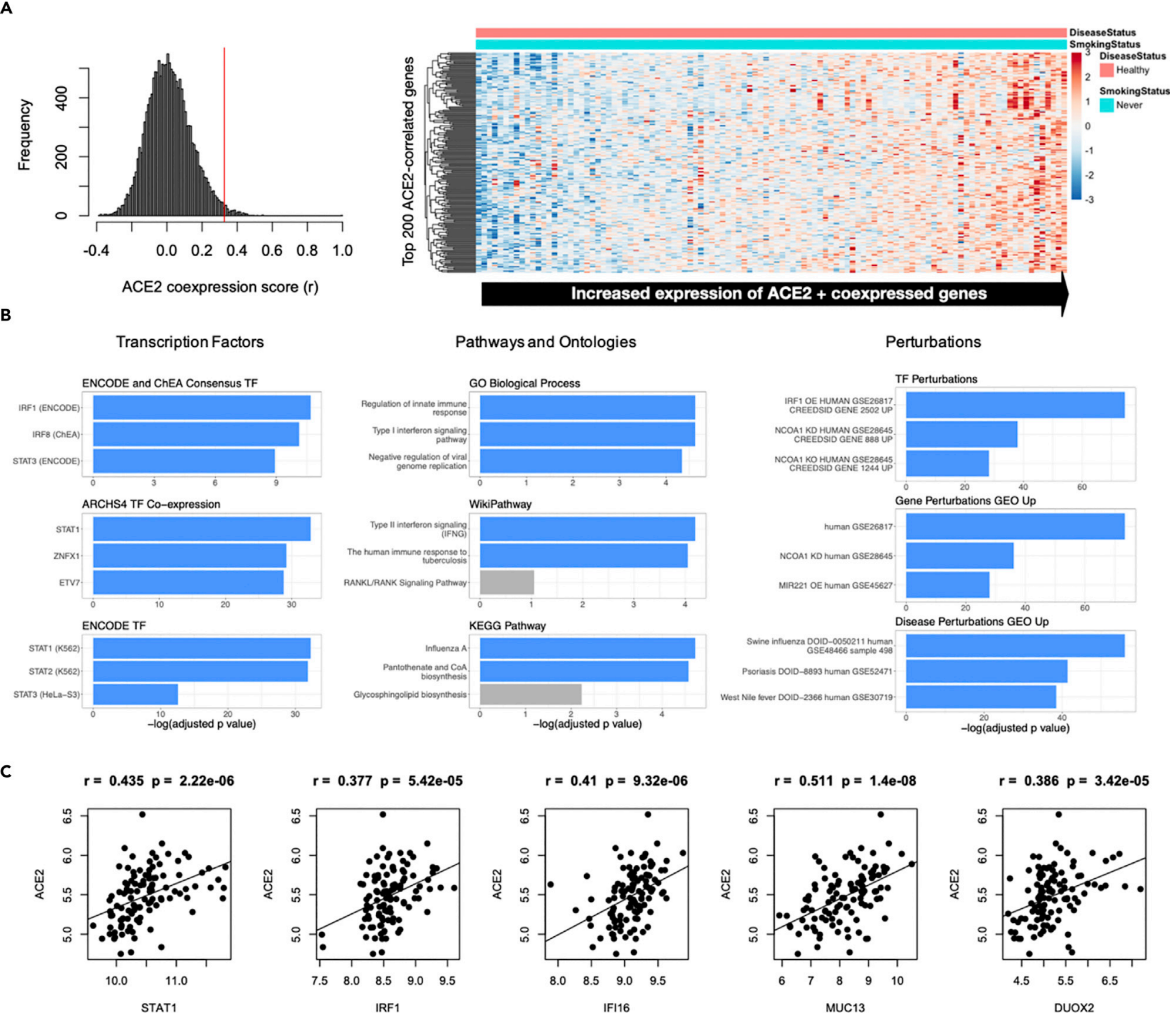
Expression and functional enrichment analysis of *ACE2* and co-expressed genes (A) Expression of top 200 *ACE2*-correlated genes (including *ACE2*) in healthy non-smokers (N = 109). (B) Functional enrichment analysis of top 200 *ACE2*-correlated genes (including *ACE2*). Terms are ranked by -log_2_(FDR-adjusted p value) for nine ontologies/groups of interest. (C) Pearson correlation of *ACE2* with important interferon-related candidate genes found to be co-expressed with *ACE2*.

To first examine patterns of baseline *ACE2* gene expression, we analyzed microarray expression datasets from a cohort of healthy, never-smokers (N = 109) (see Table S1 for accessions). In these individuals, *ACE2* was co-expressed with a gene set that is most significantly enriched in immune signaling and virus perturbations (Figure 1, Table S1). The top transcription factors associated with these genes included IRFs and STATs (e.g., *IRF1* and *STAT1*). Consistent with this finding, both *IRF1* and *STAT1* genes were also among the top 200 *ACE2-*correlated genes. Other genes that were associated with these enriched “immune-response”, and “viral response” terms, and co-expressed with *ACE2*, include *IFI16*, *IFI44*, *IFI35*, *NLRC5*, and *TLR3.* These findings suggest that in healthy never-smokers, *ACE2* may be a component of an immune signaling pathway, specifically relating to viral sensing and response and potentially mediated by IRF and STAT transcription factors.

Given the above findings on viral sensing, we next looked for the presence of these co-expressed immune response genes in an aggregated resource of COVID-19 RNA-seq studies (Butler et al., 2021; Park et al., 2022). We found that all of the genes highlighted above were also significantly upregulated in infected and diseased patients. More broadly, we also found that interferon signaling genes (e.g., *STAT1*) were upregulated in infected samples (Figure S1). This suggests that our association between *ACE2* co-expression and immune signaling in healthy microarray expression datasets also reflects the importance of immune signaling (possibly via IRF and STAT regulation) during *SARS-CoV-2* infection and disease.

During our analyses, we also detected as co-expressed with *ACE2*, *DUOX2*, a known response factor to reactive oxygen species (ROS) (Ewald, 2018). This suggests that oxidative stress may be another important mechanism that regulates *ACE2* expression (see below). Also of interest are genes that help identify cell-type-specific regulation of *ACE2*, along with its co-regulated gene network. The third top-correlated gene with *ACE2* in healthy non-smokers was *MUC13*, an epithelial mucin known to be expressed in the large intestine and trachea (Williams et al., 2001) as well as in goblet cells, which are all proposed sites of *SARS-CoV-2* replication (Gheblawi et al., 2020).

We next examined a cohort of N = 136 individuals with asthma to investigate whether the observed associations (e.g., between *ACE2* co-expression and inflammatory signaling) persisted in individuals with chronic inflammatory lung disease (Figure S2). *ACE2* co-expression in asthmatic individuals was also associated with immune signaling, antiviral responses, and IRF and STAT transcription factors. The top *ACE2*-correlated gene in asthmatics was *CD47*, which is involved in the regulation of interferon gamma. Consistent with this finding, *ACE2* and *CD47* are both co-expressed with the interferon-inducible gene *IFI44*, whose expression is regulated by IFN-γ exposure (Zeng et al., 2006)*.* Interestingly, *IFI44* has been suggested as a key target for controlling the cytokine storm-induced immunopathology observed in patients with influenza virus and high pathogenic coronavirus infections (Dediego et al., 2019). Based on our microarray expression analyses, we hypothesized that *ACE2* transcriptional regulation is associated with an immune signaling pathway involving IRF and STAT factors.

An important limitation of microarray data concerning *ACE2* is the inability to discriminate between full-length *ACE2* and the recently discovered short-length isoform *dACE2* (Onabajo et al., 2020). Therefore, the relative contribution of full-length ACE2 versus short-form dACE2 to these expression profiles remains unclear. We therefore sought more explicit, experimental interrogation of *ACE2* gene regulation by considering the *cis-*regulatory landscape of the *ACE2* locus.

### Identification of functional intronic *ACE2* regulatory elements with STAT1 and IRF1 binding sites

**Figure 2:**
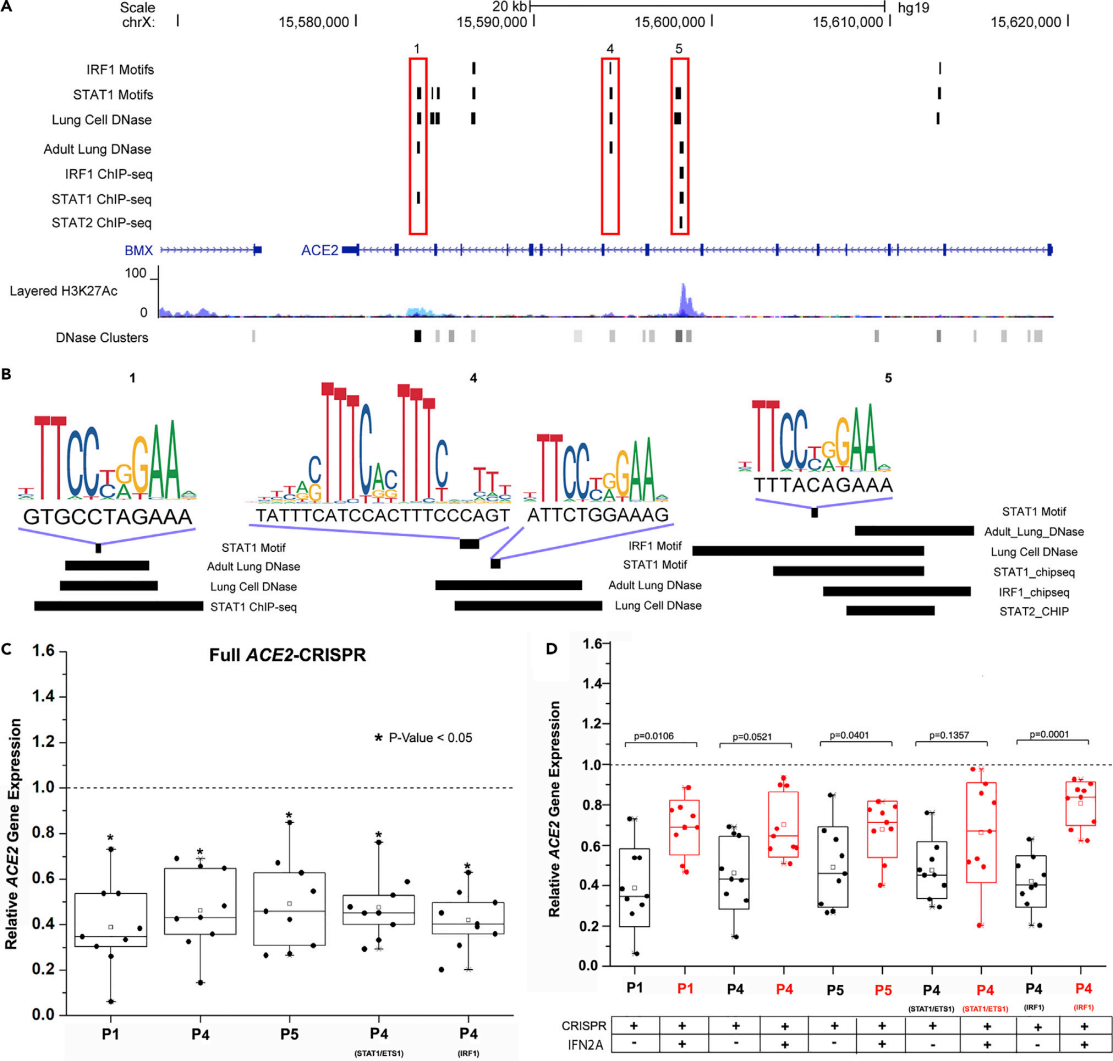
Identification of putative viral-response elements (STAT1 and IRF1 binding sites) in the *ACE2* intronic region (A) Identified transcription factor binding sites in *ACE2* intronic regions in the human genome (hg19). Three separate regions labeled 1,4, and 5 contain overlapping ChIP-seq peaks including IRF1, STAT1, and STAT2 binding sites, as well as DHS in lung cell lines indicative of open-chromatin and active transcriptional regulation. (B) DNA sequence matches to predicted IRF and STAT transcription factor binding sites in the three regions identified above, with corresponding ChIP-seq peaks indicated as horizontal bars. (C) Deletion of regions leads to decreased expression of full-length *ACE2* (n = 9; ∗p < 0.05 to respective empty vector-transfected WT cells, two-tailed Student’s T-test). Boxplots indicate upper/lower quartiles and median of experimental data. (D) Reductions in expression become attenuated when elements are deleted in the presence/absence of IFN-α treatment (n = 9; ∗p < 0.05 to respective IFN-α untreated CRISPR KO cells, two-tailed Student’s T-test). See also Table S2.

Gene expression is controlled by regulatory sequences bound by transcription factors. We next examined the regulatory region encompassed by *ACE2*, compiling chromatin-accessibility datasets (i.e., DNase-I Hypersensitivity Sites, (DHS)) from *in vitro* and adult *in vivo* lung samples from the ENCODE project (Davis et al., 2018) (Figure 2). We identified six intronic putative regulatory regions overlapping either cell-line or primary tissue DHS signals, a number of which also possess potential binding motifs for STAT1 and IRF1. We then refined this list to three intronic putative regulatory elements (Regions 1,4, and 5 in Figure 2) that overlap DHS data from both lung cell and tissue data and which contained either predicted STAT1 and IRF1 binding motifs and/or aggregated ChIP-seq datasets for each factor (see STAR Methods). These predicted factor binding motifs may be directly bound by STAT1 (Regions 1 and 5), and one possibly bound by IRF1 (Region 5). Furthermore, these putative regulatory elements were also identified in a previous study of regulatory activity in the *ACE2* locus (Lee et al., 2021).

In order to test the functionality of these three regulatory elements on *ACE2* regulation, we designed CRISPR guide RNAs (sgRNAs) to target and delete each element. We also designed sgRNAs to target predicted STAT1 and IRF1 motifs in Region 4 (Table S2). We tested our targeting strategy *in vitro* on a human lung epithelial cell line (Calu-3). To rule out potential off-target effects, we first confirmed that transfection of sgRNA plasmids did not disrupt the expression of nearby genes. Expression levels of nearby *TMEM-27* and *BMX1* were not significantly altered with deletion of any element or putative binding site (Table S2). Using full-length isoform-specific primers, we next assessed levels of full-length *ACE2* transcripts using qPCR in wild type and CRISPR-deleted cells. We found that full-length *ACE2* expression was significantly decreased with deletions of each individual element, as well as the targeted binding sites within Region 4 (Figure 2C and Table S2). A recent study identified that the dACE2 isoform is regulated upon *SARS-CoV-2* infection (Onabajo et al., 2020). Interestingly, using primers specific to *dACE2*, we found that its transcript levels were also significantly decreased in our deletion experiments, and to a greater degree compared to full-length *ACE2* (Table S2). These results indicate that, in the absence of additional perturbation (i.e., above transfection), each of the three candidate intronic regulatory sequences we tested acts as an enhancer specifically for *ACE2*.

Our bioinformatic analyses of RNA-expression datasets and subsequent motif/ChIP-seq scans suggest that *ACE2* expression is regulated by an immune signaling pathway, possibly through STAT1 and IRF1 binding activity intronic to the *ACE2* locus. We therefore tested the effects of deleting these putative immune-responsive elements and specific binding sites in the context of immune signaling. Type I interferons (IFNs), such as IFN-α are our first line of defense against invading viruses (Stanifer et al., 2020). We used IFN-α treatment to induce intracellular immune signaling pathways that would occur during viral infections (Taniguchi and Takaoka, 2002) (see STAR Methods). We first performed this experiment on wild-type cells and found that this treatment led to a moderate increase of full-length *ACE2* transcripts only after 48H (Figure S3A), with *dACE2* levels increasing strongly and significantly at both time points, consistent with previous studies (Lee et al., 2021; Onabajo et al., 2020) (Table S2). We independently confirmed this finding at the protein level (Figure S3B), and further found that additional potent inducers of immune signaling, such as poly(I:C) treatment and direct infection with *SARS-CoV-2*, did not lead to significant upregulation of full-length ACE2 at the protein level.

We next performed IFN-α treatment in the context of enhancer deletion. We observed a significantly decreased effect of CRISPR element deletion on full-length *ACE2* gene expression reduction with IFN-α stimulation compared to expression changes in the absence of stimulation (Figure 2D and Table S2). This was observed across the majority of our element and sub-element (i.e., motif) deletions. This attenuated downregulation was also observed for *dACE2* across stimulation-deletion experiments (Table S2). These findings suggest that these enhancer elements may be in part responsive to immunological stimulation (via IFNs) and play a role in a more complicated, potentially redundant, regulatory mechanism for *ACE2* expression (see Discussion).

### *ACE2* gene expression in lung epithelial cells is correlated with smoking and COPD disease status and associated with an *NRF2* antioxidant response

While much of the initial medical literature associated with *SARS-CoV-2* patient demographics suggest a link between smoking status and disease severity (Alqahtani et al., 2020; Patanavanich and Glantz, 2020; Vardavas and Nikitara, 2020), more recent studies have cast doubts as to the strength and significance of this relationship (Cattaruzza et al., 2020; Lippi and Henry, 2020). The relationship between smoking history and respiratory viral infection disease severity has been suggested to be more complicated (Zhao et al., 2020). It is also worth noting that ACE2, in addition to being the primary receptor for *SARS-CoV-2* infection (Wang et al., 2020; Zhou et al., 2020), serves an important biological role in multiple tissues (Gheblawi et al., 2020), and is present in lung epithelium (Aguiar et al., 2020; Hikmet et al., 2020). Thus, shifts in basal expression levels of this protein, especially over time, may contribute to lung dysfunction in an indirect, more complex manner than can be measured using metrics such as COVID-19 disease severity. Given this possibility, we next assessed expression patterns of bronchial brushing datasets from current and previous smokers, focusing on *ACE2* and other co-expressed genes (e.g., *DUOX2*).

**Figure 3:**
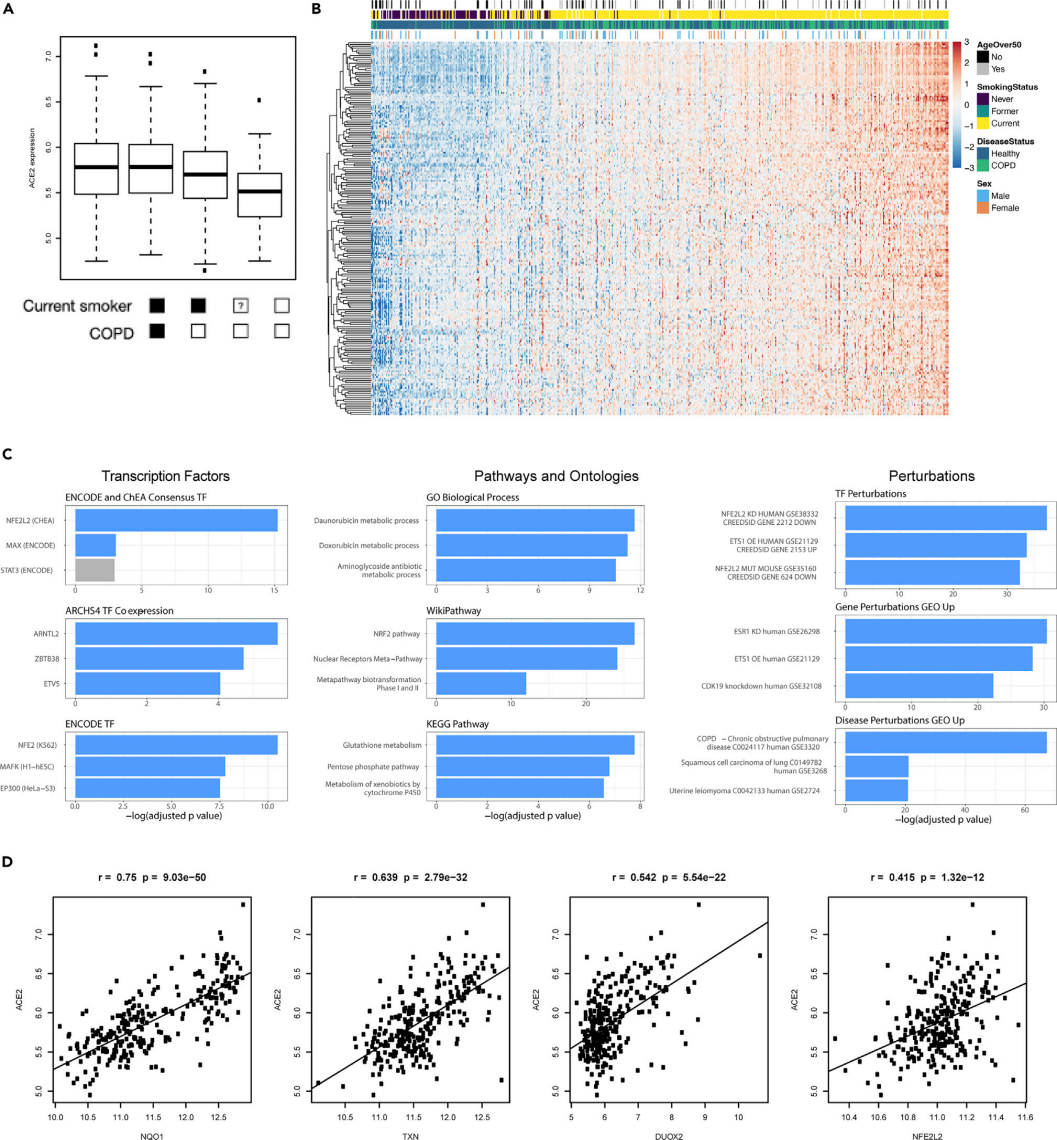
Expression and functional enrichment analysis of *ACE2* and co-expressed genes in smokers and individuals with COPD (A) Analysis of relative *ACE2* expression with respect to smoking status and COPD diagnosis. Boxplots indicate upper/lower quartiles and median of expression data. (B) Expression of top 200 *ACE2*-correlated genes (including *ACE2*) in individuals with various smoking status and COPD diagnosis (N = 159). (C) Functional enrichment analysis of top 200 *ACE2*-correlated genes (including *ACE2*). Terms are ranked by -log_2_(FDR-adjusted p value) for nine ontologies/groups of interest. (D) Pearson correlation of *ACE2* with important interferon-related candidate genes found to be co-expressed with *ACE2*.

We analyzed a dataset of 159 healthy current smokers versus healthy former smokers, and identified the top 200 *ACE2*-correlated genes (Figure 3, Table S1). Expression patterns for *ACE2* suggest current smoking status is associated with increased *ACE2* levels, consistent with previous observations (Brake et al., 2020; Cai et al., 2020; Leung et al., 2020a, 2020b), and that this also accounts for the increased *ACE2* in patients with COPD (Figure 3A). Expression patterns for *ACE2*-correlated genes alone were able to effectively distinguish smokers from non-smokers (Figure 3B). Functional enrichment analysis showed that in this dataset, genes co-expressed with *ACE2* are significantly associated with the NRF2 pathway, oxidative stress, glutathione metabolism, and TGF-β regulation of the extracellular matrix. *NRF2* is a key transcription factor that regulates the oxidative stress response in the lung (Gebe et al., 2010; Rangasamy et al., 2004). Consistent with this, according to both ChIP-seq data and gene expression perturbation data, *NRF2* (*NFE2L2*) was the top transcription factor identified as a likely regulator of these genes; for example, the NRF2-regulated antioxidant gene *NQO1* was the fourth ranked *ACE2*-correlated gene in this dataset. *ACE2*-correlated genes also overlapped significantly with genes upregulated by the transcription factor ETS1 (GSE21129) (Verschoor et al., 2010); ETS1 is an important regulator of ROS (reactive oxygen species) in response to angiotensin II, linking it to ACE2 function (Ni et al., 2007). Moreover, *ETS1* expression is induced by ROS exposure through an antioxidant response (Wilson et al., 2005). Thus, *ACE2* expression in smokers appears to be associated with oxidative stress gene regulation, likely mediated by NRF2 and ETS1.

To verify these trends, we repeated the same analyses with a second cohort dataset from a different microarray platform associated with 345 healthy smokers versus healthy non-smokers (Figure S4). Notably, this dataset consists predominantly of younger individuals (age <50), whereas the first dataset includes predominantly older individuals (>50). *ACE2* co-expression patterns were highly correlated between the two independent datasets providing support that these are robust signals. As with the first analysis, genes co-expressed with *ACE2* showed the strongest associations with *NRF2* gene targets. Moreover, *NRF2* and *ETS1* formed the top three overlapping datasets according to enrichments for transcription factor perturbation datasets (Figure S4C). This dataset also included a larger number of patients with COPD; that we observed similar patterns of *ACE2* expression and co-expressing genes in this analysis may suggest similar effects on smoking and disease status on *ACE2* regulation (see Discussion).

### Identification of functional intronic *ACE2* regulatory elements with possible antioxidant-response element (ARE) activity

**Figure 4:**
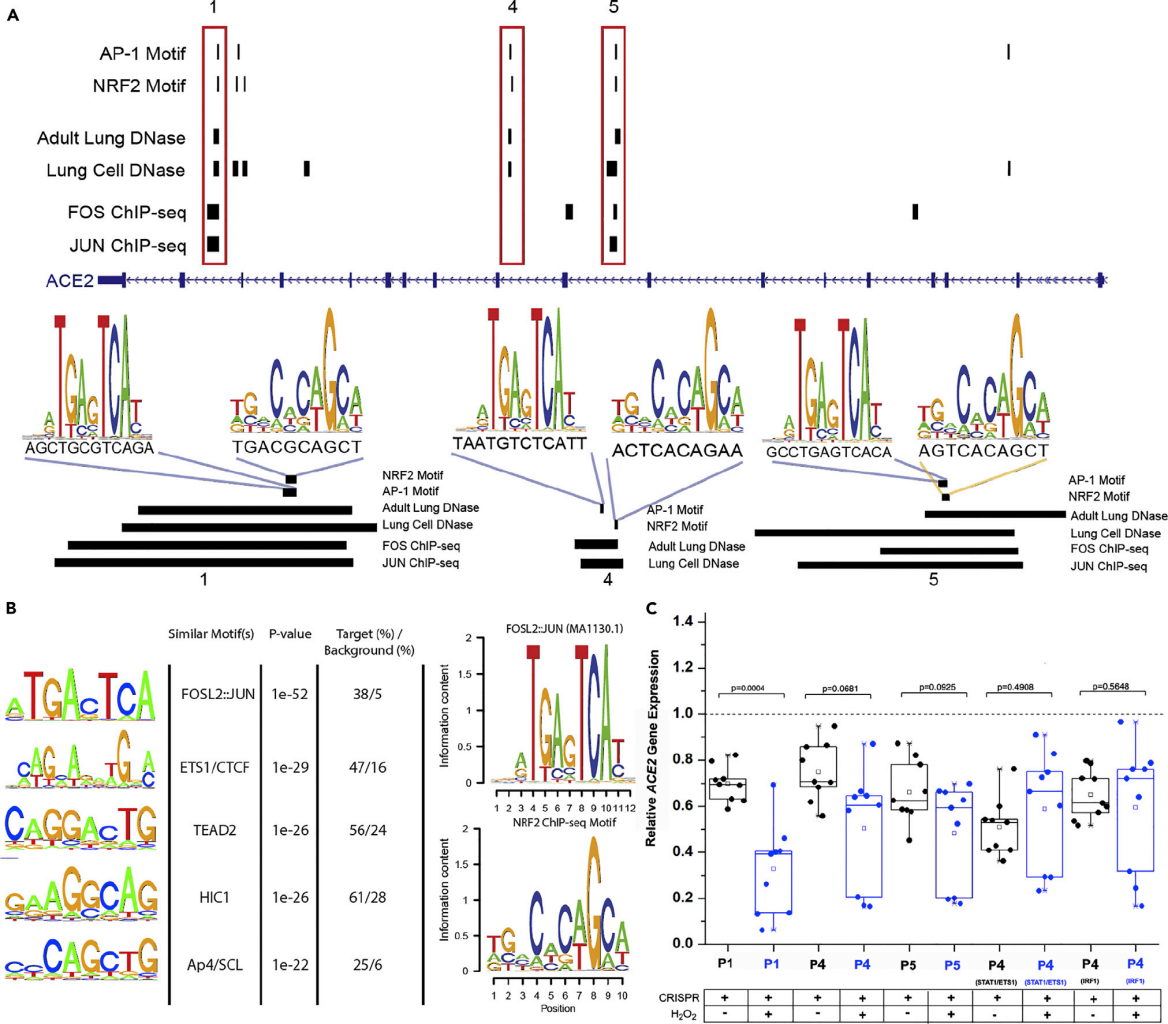
Putative antioxidant response elements (AREs) in the *ACE2* regulatory region (A) Predicted AP-1 and NRF2 binding sites in *ACE2* intronic regions in the human genome. Three separate regions labeled 1, 4, and 5 (red boxes) contain overlapping ChIP-seq peaks including FOS/JUN binding sites, as well as DNase hypersensitivity peaks in adult lung tissue and cell line samples indicative of open-chromatin and active transcriptional regulation. Shown below are DNA sequence matches of predicted AP-1 and NRF2 transcription factor binding sites in the three regions identified above, with corresponding ChIP-seq peaks indicated as horizontal bars. (B) (Left) Top statistically over-represented motifs in *ACE2* non-coding regulatory regions and their top matches to known transcription factor binding preferences. The top two binding motifs identified bear strong resemblance to FOSL2:JUN (AP1) and (ETS1/CTCF). (Right) The JASPAR-database FOSL2:JUN binding motif (MA1130.1) was enriched in intragenic *ACE2* elements. Also shown is an NRF2 binding motif defined using ChIP-seq data. Both motifs in (b) were used to scan intragenic *ACE2* element sequences in (a). (C) Deletion of regions leads to decreased expression of full-length *ACE2* in the presence/absence of oxidative stress (blue boxes). P-values indicate results of two-tailed Student’s T-test. Boxplots indicate median, upper, and lower quartiles of expression data. See also Table S2.

Our microarray data analyses led to a predicted association between oxidative stress and *ACE2* levels, which prompted us to consider the existence of antioxidant-response elements (AREs within the *ACE2* locus (Figure 4A). We performed an unbiased analysis of the regulatory regions intronic to *ACE2* (Figure 4B). Our analysis identified AP-1 as the top enriched transcription factor binding site in the locus, suggesting that AP-1 may be an additional regulator of *ACE2.* Klatt and colleagues have shown AP-1-*c*-Jun subunit binding to DNA is dependent on the cellular GSH/GSSG ratio, a marker of cellular ROS levels (Klatt et al., 1999). Looking at the top *ACE2*-correlated genes across all datasets, we identified significant co-expression between *ACE2* and *FOSL2*, as well as *ACE2* and *JUN.* This suggests that AP-1 may be a transcription factor involved in oxidative stress-mediated regulation of *ACE2* levels.

Given the observed motif enrichments for AP-1 and NRF2 transcription factor binding sites in the *ACE2* locus, we next looked at the individual predicted motif hits for AP-1 and NRF2 factors within the six putative regulatory regions defined above (Figure 4B). Three of these enhancers (Regions 1, 4, and 5) contained predicted AP-1 and NRF2 binding sites and were active in both *in vivo* and *in vitro* lung datasets. Two of these regions (Region 1 and 4) also overlapped ChIP-seq datasets for FOS and JUN factors, important co-factors associated with AP-1 complex (Rauscher et al., 1988) and NRF2 (Jeyapaul and Jaiswal, 2000) enhancer binding, respectively. We next sought to delete these elements in the context of oxidative stress, with the expectation that, if these elements act as AREs, that the effects of deletion should be magnified during an ROS response.

We first examined *ACE2* transcript levels after exposing wild-type Calu-3 cells to hydrogen peroxide (0.5mM), a potent ROS (Boardman et al., 2004) (see STAR Methods). We found that exposure to hydrogen peroxide led to significant decreases in expression of both full-length *ACE2* and *dACE2* (Figure S5, Table S2). Previous mouse studies have also demonstrated a downregulation of *ACE2* levels and activity following acute ROS exposure (Fang et al., 2019). However, *ACE2* levels are upregulated with chronic oxidative stresses (Gebe et al., 2010; Smith et al., 2020); this may suggest a more complicated regulation of *ACE2*, possibly as a function of time (see Discussion).

We next deleted each regulatory element and assessed *ACE2* expression in the presence/absence of hydrogen peroxide treatment (see STAR Methods). We observed a significant decrease in basal levels of full-length *ACE2* transcripts for the majority of regions/sites deleted in the absence of external stimulus (Table S2). In the context of exogenous oxidative stress, we observed a further significant decrease only for Region 1, while all others trended downward (Table S2). For this first region, the magnitude of downregulation was significantly greater under oxidative stress when compared to the unstimulated deletion change (Figure 4C), while for other regions magnitudes were similarly larger despite the lack of significance (potentially due to the increased variability in expression observed in ROS-stressed cells). This first region contains predicted NRF2 and AP-1 motifs, and also overlaps with both FOS and JUN ChIP-seq signals, possibly explaining the increased effect of deletion under ROS conditions. Finally, we again saw these differences to be accentuated when considering levels of the *dACE2* transcript (Table S2).

## Discussion

Regulation of *ACE2* expression at the transcriptional level may impact susceptibility to viral infection. Subsequently, changes to *ACE2* expression during viral infection can lead to an imbalance in renin-angiotensin system (RAS) signaling contributing to the manifestation of clinical symptoms such as excessive inflammation (Mahmudpour et al., 2020), myocardial injury (Clerkin et al., 2020), and lung injury (Ni et al., 2020b). It has recently been suggested that dysregulation of the RAS system during initial infection may be partially responsible for the activation of the cytokine storm observed in some severe cases of COVID-19 (Mahmudpour et al., 2020), wherein the loss of surface ACE2 promotes the release of inflammatory cytokines via enhanced Ang II signaling. Elevated Ang II has also been suggested to mediate consequences of RAS imbalance tied to COVID-19 (Lanza et al., 2020), including severe hypoxia and lymphopenia. These findings indicate that targeting transcriptional inhibition of *ACE2* expression may be a therapeutic avenue for prevention of severe COVID-19 infection (Qiao et al., 2020), while counteracting infection-induced *ACE2* downregulation may act as a therapeutic treatment to reduce disease severity (Chatterjee and Thakur, 2020; Zhang et al., 2020b).

In the first part of our study, we utilized microarray expression datasets from healthy non-smokers and identified genes whose expression patterns significantly correlated with that of *ACE2*. Groups of correlated genes may suggest shared upstream regulators; gene set enrichment analyses indicated that *ACE2* and correlated genes may be under the control of immune signaling pathways integrating on the STAT and IRF families of transcription factors—namely, STAT1 and IRF1. These results were also observed when performing a separate analysis on asthmatics individuals (Figure S2). Moreover, expression data from COVID-19-infected samples further substantiated this association with immune signaling pathways (Figure S1). Interestingly, a recent study of microarray datasets from *SARS-CoV-2* infected cells suggested the importance of JAK-STAT signaling (Luo et al., 2021). In that study, the authors found that JAK-STAT components (e.g., *STAT1*) were co-expressed with *ACE2* in *SARS-CoV-2*-infected cell lines, and suggested that ACE2 could act to regulate JAK-STAT activity. Given our computational and experimental findings, we suggest that immune signaling pathways such as JAK-STAT act upstream to regulate *ACE2*. This hypothesis has been further corroborated by an independent study, which found that the transcriptional response of *ACE2* (as well as *dACE2*) to interferon stimulation was mitigated by pharmacological inhibition of JAK (Lee et al., 2021). Interestingly, this study also found that the expression of genes identified in our correlated expression analysis, including *STAT1*, *IFI44*, and *IRF1*, were also under control of immune signaling pathways involved in interferon response (Lee et al., 2021).

Given our computational findings, we considered *cis*-regulatory elements within the *ACE2* locus which may be proximate mediators of this immune-response regulatory mechanism. After identifying six such intronic regions, we prioritized and then experimentally deleted three putative intronic enhancers, containing predicted STAT1 and IRF1 binding motifs. Deletion of these three elements (Regions 1, 4, and 5) separately led to a consistent downregulation of *ACE2* transcripts. The downregulation of *ACE2* upon deletion, relative to mock-transfected controls, suggests that these enhancers may contribute to basal expression both individually but possibly collectively. This latter possibility could not be examined because experimental deletion in tandem of enhancers spanning separate introns would likely generate *ACE2* loss-of-function contexts as well as complex gene/regulatory element interactions at the locus. Interestingly, performing individual deletion of each enhancer in the context of immune stimulation, we did observe a significant attenuation of the downregulation caused by our deletions, while immune stimulation in wild-type cells caused moderate changes to *ACE2* expression (Figure S3). These results corroborate our previous findings that *SARS-CoV-2* infection does not significantly upregulate transcript levels of *ACE2* in Calu-3 cells in spite of significant increases in type I and type III IFNs, as well as upregulation of known interferon-stimulated genes (e.g., *IFIT1*, *IRF7*, and *OAS2*) (Banerjee et al., 2021). The observed attenuation effect may suggest an upper-threshold or “saturation” of *ACE2* expression from baseline, such that immune signaling does not lead to a substantial increase. However, following deletion of these putative enhancers, proximate regulators of immune signaling (e.g., STAT1 and IRF1) acting elsewhere in the *ACE2* locus (e.g., at the promoter level (Ziegler et al., 2020)) may be able to compensate for the loss/reduction in enhancer activity. Further experimentation (e.g., using a viral-infection model system) into this complex regulatory system may elucidate the role that these enhancers play in upregulating *ACE2* during an immune response.

An understanding of the mechanisms regulating *ACE2* expression during viral infection is important from a disease-pathology point of view, given that this may inhibit the protective effects of ACE2 activity. In addition, understanding the regulatory mechanisms controlling *ACE2* expression prior to viral exposure may be of equal importance from the perspective of disease prevention, given that baseline levels of *ACE2* in high-exposure tissues (e.g., lung) may modify viral susceptibility (Devaux et al., 2020).

It has been suggested, though not conclusively shown, that chronic smokers are at elevated risk to both *SARS-CoV-2* infection as well as severe disease (Alqahtani et al., 2020; Cattaruzza et al., 2020; Patanavanich and Glantz, 2020). This follows with previous studies of other coronaviruses, e.g., *MERS-CoV*, for which epidemiological evidence does suggest smoking status as a key risk factor (Arcavi and Benowitz, 2004; Park et al., 2018). In terms of increased susceptibility, it may be that smokers have elevated baseline *ACE2* expression in lung tissues, increasing the likelihood that *SARS-CoV-2* may bind their target receptors (Qiao et al., 2020). In the second part of our study, we analyzed microarray expression data from two independent datasets consisting of current smokers, non-smokers, and patients with COPD. Considering the expression of *ACE2*, we also observed previously reported increases in baseline expression within smokers (Qiao et al., 2020; Smith et al., 2020). With this, as well as genes showing similar transcriptional behaviors, we identified enrichments for oxidative stress-response pathways, including transcriptional regulators such as NRF2 and the AP-1 complex. These signals are indicative of another potential regulatory mechanism acting on the *ACE2* locus, and are expected given the chronic oxidative stresses experienced by habitual smokers and patients with COPD (Pierrou et al., 2007).

Looking again at the *ACE2* locus, we found enrichment in open-chromatin regions (putative enhancers) for DNA sequences bearing similarity to known FOSL2:JUN binding motifs, further suggesting the regulatory effects of oxidative-response signaling at this locus. We therefore performed another set of targeted deletion experiments of intronic enhancers most likely to behave as antioxidant-response elements (AREs) (e.g., contain NRF2 motifs, AP-1 ChIP-seq signals, etc.). Performing these deletions in the context of exogenous oxidative stress yielded a substantial decrease in *ACE2* expression for the first element tested (Region 1), with a fold-change decrease below that observed in wild-type cells following treatment.

We suggest that this putative ARE, and potentially others which trend in the same direction, act to counter the inhibitory effects of oxidative stress on *ACE2* expression*,* which has been previously observed in a mouse model of hyperoxia (Fang et al., 2019), preventing a more deleterious loss of ACE2 protein following acute exposure. ACE2 plays an important role in mitigating acute oxidative stress (Xia et al., 2011; Zheng et al., 2014), particularly in the context of cardiovascular and lung disease (Rabelo et al., 2011; Shenoy et al., 2011). We further propose that the repression of *ACE2* upon acute oxidative stress, when repeated on the order of decades in chronic smokers, may lead to an “over-compensation” of baseline *ACE2* expression—establishing higher levels of ACE2 protein to protect lung tissues from further damage. This process could be mediated by a number of oxidative stress-response mechanisms; in particular, our observed enrichments for NRF2-regulated genes co-expressed with *ACE2*, along with the presence of NRF2 motifs within intronic *ACE2* enhancers, follow with the protective role of NRF2 signaling induced in response to cigarette smoke (Ma, 2013). Furthermore, a mouse-model study of cigarette smoke found significant increases in ACE2 activity only after three weeks of exposure (Hung et al., 2016), while additional studies have found dose-response effects with increased treatment time (Gebe et al., 2010; Liu et al., 2021). Human smokers also exhibit a dose-response effect of *ACE2* expression with increasing pack-years (Smith et al., 2020). However, we acknowledge the speculative nature of this proposed over-compensating effect and note the importance of additional experimental testing. While the links between smoking status and COVID-19 severity are controversial, the link between COPD status and COVID-19 severity may be clearer (Leung et al., 2020a, 2020b; Olloquequi, 2020; Zhao et al., 2020). More generally, it has been suggested that the detrimental effects of smoking, most notably attenuation of antiviral innate immune responses (Sopori, 2002), can increase susceptibility to pathogen infection (Arcavi and Benowitz, 2004).

In summary, here, we explored the regulatory mechanisms which may act on the *ACE2* locus in the context of both immune stimulation as well as oxidative stress, leading us to identify two putative pathways which may mediate this transcriptional regulation. It is important to note that these pathways are not mutually exclusive; the links between immune signaling and oxidative stress are well established (Rahman et al., 1996), and this is particularly true for ACE2 given its biological role in RAS regulation (Gheblawi et al., 2020; Wang et al., 2020). We suggest that further experimental testing is warranted to confirm these predicted mechanisms, and furthermore to develop potential strategies taking advantage of this knowledge to modify susceptibility and disease severity of coronavirus infections, particularly *SARS-CoV-2*.

### Limitations of the study

The microarray analysis performed is limited to the detection of transcripts for which corresponding probes exist on the given chip, meaning that splice isoforms and rare variants are unlikely to be detected. This limitation impacts our study by preventing the detection of dACE2 and differentiation between this isoform and the canonical ACE2 within the sample population. Given that our identified intronic elements span multiple different exons in *ACE2*, it is extremely difficult to generate serial deletions in *cis* of these enhancers without generating any *ACE2* loss-of-function scenarios. Thus, we cannot comment on the potential combinatorial effects of different intronic *ACE2* regulatory elements to regulating expression.

## STAR★Methods

### Key resources table

**Table:** 

REAGENT or RESOURCE	SOURCE	IDENTIFIER
**Antibodies**		

1:1000 mouse anti-SARS/SARS-CoV-2 N	ThermoFisher Scientific	MA5-29981; RRID: AB_2785780
1:1000 rabbit anti-beta-actin	Abcam	ab8227; RRID: AB_2305186
mouse anti-ACE2	R&D Systems	MAB933; RRID: AB_2223153
1:5000 donkey anti-rabbit 800	LI-COR Biosciences	926-32213; RRID: 621848
1:5000 goat anti-mouse 680	LI-COR Biosciences	Catalogue number: 925-68070; RRID: AB_2651128

**Bacterial and virus strains**		

SARS-CoV-2	Clinical isolate	SARS-CoV-2/SB3
Biological samples		

**Chemicals, peptides, and recombinant proteins**		

Human recombinant interferon alpha 2	OriGene Technologies	TP723881
Recombinant human IFNβ1	ThermoFisher Scientific	R69007

**Critical commercial assays**		

Direct-zol RNA Miniprep kit	Zymo Research Corporation	R2072
Applied Biosystems Power SYBR master mix	ThermoFisher Scientific	4368577
Lipofectamine 2000	Invitrogen	D5210
SuperScript III First Strand cDNA Synthesis Reaction kit	Life Technologies	18090010

**Deposited data**		

Human reference genome NCBI build 37, GRCh37	Genome Reference Consortium	https://www.ncbi.nlm.nih.gov/projects/genome/assembly/grc/human/
See Table S1 for list of accessions for publicly-available microarray expression datasets used in this study.		
NRF2 ChIP-seq dataset	Chorley et al., 2012	GEO GSE37589
ENCODE DNase-seq datasets used	Yue et al., 2014	ENCFF271JAF, ENCFF331SYD, ENCFF681UOZ, ENCFF165ZIA, ENCFF334RSR, ENCFF338GII, ENCFF446FTN, ENCFF460ZFL, ENCFF546QUZ, ENCFF644XOI, ENCFF957JQC
JASPAR Database	Mathelier et al., 2016	https://jaspar.genereg.net/
ChIP-ATLAS	Oki et al., 2018	https://chip-atlas.org/
Supplemental information	This study; Mendeley Data	https://doi.org/10.17632/wmv6f24xm2.1

**Experimental models: Cell lines**		

Calu-3	ATCC	HTB-55

**Oligonucleotides**		

See Table S2 for all PCR primers.		
See Table S2 for all oligonucleotides used in CRISPR experiments.		

**Recombinant DNA**		

PX458 plasmid	Addgene	101731

**Software and algorithms**		

Bedtools version 2.26.0	Quinlan and Hall, 2010	https://bedtools.readthedocs.io/en/latest/
HOMER version 4.10	Heinz et al., 2010	http://homer.ucsd.edu/homer/
R base version 3.6.3	R Development Core Team, 2008	https://www.r-project.org/
COVID-19 Genes	Butler et al., 2021, Park et al., 2022	https://covidgenes.weill.cornell.edu/
GEOquery version 2.52.0	Sean and Meltzer, 2007	https://www.bioconductor.org/packages/release/bioc/html/GEOquery.html
sva version 3.32.1	Leek et al., 2012	https://www.bioconductor.org/packages/release/bioc/html/sva.html

### Resource availability

#### Lead contact

Further information and requests for resources should be directed to and will be fulfilled by the lead contact, Terence D. Capellini (tcapellini@fas.harvard.edu).

#### Materials availability

This study did not generate new unique reagents.

### Method details

#### *ACE2* co-expression and functional enrichment analysis with public microarray data

Public microarray experiments using Affymetrix chips (HuGene-1.0-st-v1 and HG-U133 Plus 2) on airway epithelial cell samples were obtained from the NCBI Gene Expression Omnibus (GEO) database. This resulted in a total of 1859 individual samples from 33 different experiments (See Table S1). Within this dataset, disease status (Healthy: 504, COPD: 338, Asthma: 136) and/or smoking status (Never: 409, Former: 139, Current: 956) information was included for 1716 samples. For all datasets, raw intensity values and annotation data were downloaded using the *GEOquery* R package (version 2.52.0) (Sean and Meltzer, 2007) from the Bioconductor project. Probe definition files were downloaded from Bioconductor and probes were annotated using Bioconductor’s *annotate* package. All gene expression data were unified into a single dataset that was then RMA-normalized, and only genes present in both of the Affymetrix platforms (N = 16,105) were kept for subsequent analyses. Correction of experiment-specific batch effects was performed using the ComBat method implemented using the *sva* R package (version 3.32.1) (Leek et al., 2012).

Top *ACE2* co-expressed genes were identified based on the 200 highest Pearson correlation (*r*) values to *ACE2*. Heat maps for top 200 *ACE2*-correlated genes across samples were generated with the *pheatmap* R package (version 1.0.12). For display only, expression values were row-normalized (across gene) using the ‘scale’ function in base R, and converted to Z-scores. For heatmap coloring, a “ceiling” and “floor” of +3 and −3 was applied to the Z-scores. Histograms and *ACE2* correlation scatterplots were generated with the *base* R package (version 3.6.3) (R Development Core Team, 2008). Gene lists are available in Table S1.

The top 200 *ACE2*-correlated genes were analyzed using Enrichr(71) to identify enriched pathways and functional ontologies. Terms were ranked within ontologies by FDR-adjusted p value (calculated by Enrichr by running the Fisher exact test for random gene sets in order to compute a mean rank and standard deviation from the expected rank for each term in the gene set library) and the top 3 terms for ontologies of interest were selected. Functional enrichment bar plots were generated with the *ggplot2* R package (version 3.2.1).

To parse transcriptional datasets from SARS-CoV-2 infected samples, we made use of the aggregated meta-analysis dataset and analyses performed by Butler et al. (2021) and Park et al. (2022) (Butler et al., 2021; Park et al., 2022). We queried the ‘COVID19 Genes’ online interface hosted by the Mason lab at Cornell University (https://covidgenes.weill.cornell.edu/) for immune-response genes detailed in our results section (those with strong correlated expression with ACE2), finding that all of these genes were significantly elevated in CoV-2 infected samples relative to their respective dataset controls. Given our finding of interferon signaling as an enriched pathway within our set of top 200 ACE2-correlated genes, we used the ‘Pathway Enrichment’ tool from the web interface, using the term ‘REACTOME_INTEFERON_SIGNALING’. The heatmaps shown in Figure S1 were generated from the ‘Pathway Heatmap, WCM NP’ and ‘Pathway Heatmap, WCM Autopsy Lung’ output tabs.

#### *ACE2* regulatory region analyses

ENCODE (Yue et al., 2014) DNase-seq datasets were obtained for adult lung (File accessions: ENCFF271JAF, ENCFF331SYD, ENCFF681UOZ) and primary cell (ENCFF165ZIA, ENCFF334RSR, ENCFF338GII, ENCFF446FTN, ENCFF460ZFL, ENCFF546QUZ, ENCFF644XOI, ENCFF957JQC) samples as processed hg19 bed-formatted files. Called peaks (putative regulatory elements) were subsequently intersected within lung/cell sets using *bedtools* (Quinlan and Hall, 2010) (version 2.26.0), requiring that a peak be replicated in at least two samples for retention. To capture a broader regulatory region around the *ACE2* gene, peaks falling within 1MB upstream/downstream of the *ACE2* promoter were collected and pooled across lung/cell sets. Human hg19 reference sequences were obtained for these elements using UCSC (Kent et al., 2002).

Sequences were subsequently used for *de novo* motif analysis using HOMER (version 4.10) (Heinz et al., 2010) using a 10x random shuffling as a background set. *De novo* motifs were compared to a vertebrate motif library included with HOMER which incorporates the JASPAR (Mathelier et al., 2016) database. Matches are scored using Pearson’s correlation coefficient of vectorized motif matrices (PWMs), with neutral frequencies (0.25) substituted for non-overlapping (e.g., gapped) positions. Best-matching motif PWMs obtained from this analysis are shown in Figure 4B. The highest-rank motif bore close similarity to the *FOSL2::JUN* PWM from the JASPAR database (MA1130.1). This reference PWM was subsequently used for motif scanning. The AME program (McLeay and Bailey, 2010), part of the MEME-Suite (Bailey et al., 2009), was also used with these sequences to look for enrichments of known transcription factor (TF) motifs. Focusing on elements intragenic to *ACE2* as more proximate candidate regulatory regions, these reference sequences were also tested for enriched motifs using AME. The *FOSL2::JUN* (MA1130.1) PWM was used to scan these sequences using TFBSTools (Tan and Lenhard, 2016) (version 1.24.0). Hits of minimum sequence scores of 80% were retained, and subsequently filtered for Benjamini-Hochberg adjusted p value <0.05. For illustrative purposes, the best-scoring hit for each hit-containing element was selected. JASPAR-database motif matrices were also obtained for *STAT1* (MA0137.3), and *IRF1* (MA0050.2) and similarly used to scan intragenic *ACE2* elements using TFBSTools as described. Given the observed expression data for *NRF2*, a ChIP-seq dataset for this factor (GSE37589) (Chorley et al., 2012) was downloaded as called hg18 peaks. These were lifted-over to hg19 using the UCSC liftOver utility (Kent et al., 2002) with sulforaphane and vehicle-treatment datasets pooled and merged for a final set of 919 peaks. Reference hg19 sequences were obtained for these peaks and used with HOMER to define an *NRF2 de novo* motif; the resulting PWM was subsequently used with TFBSTools to scan the putative *ACE2* intragenic regulatory sequences as described above. ChIP-seq data for indicated factors (*IRF1*, *STAT1*, *STAT2*, *FOS* and *JUN*) were obtained from ChIP-ATLAS (Oki et al., 2018) as an aggregate across all cell types using a significance threshold of 50 (*q* value < 1 × 10^−5^). Peak files (hg19) were sorted and merged for overlapping peaks using bedtools. DNase, ChIP-seq, and motif hit tracks were loaded into the UCSC Genome Browser (Karolchik et al., 2013) for visualization.

#### *In-vitro* experimental validation studies

##### Poly(I:C) transfection and IFN treatment

Calu-3 cells were mock transfected with 4 μL of lipofectamine 3000 (ThermoFisher Scientific) in Opti-MEM (ThermoFisher Scientific) only or transfected with 100 ng of poly(I:C) (InvivoGen) for 6 h. Recombinant human IFNβ1 was generated using *Drosophila* Schneider 2 (S2) cells following manufacturer’s recommendation and by using ThermoFisher Scientific’s *Drosophila* Expression system (ThermoFisher Scientific). Recombinant IFNβ1 was collected as part of the cell culture supernatant from S2 cells and total protein was measured using Bradford assay. Total protein concentration was used for subsequent experiments. To demonstrate that S2 cell culture media did not contain non-specific stimulators of mammalian antiviral responses, we also generated recombinant green fluorescent protein (GFP) using the same protocol and used the highest total protein concentration (2 mg/mL) for mock treated cells. SARS-CoV-2 infected cells were treated with supernatant containing IFNβ1 or GFP for 6 h.

#### SARS-CoV-2 infection

Clinical isolate of SARS-CoV-2 (SARS-CoV-2/SB3) was propagated on Vero E6 cells and validated by next generation sequencing (Banerjee et al., 2020a). Virus stocks were thawed once and used for an experiment. A fresh vial was used for each experiment to avoid repeated freeze-thaws.

#### Immunoblots

Calu-3 cells were seeded at a density of 3 x 10^5^ cells/well in 12-well plates. Cells were infected with SARS-CoV-2 at an MOI of 1. Control cells were sham infected. Twelve hours post incubation, cells were transfected or treated with poly(I:C) or IFNβ, respectively for 6 h. Cell lysates were harvested for immunoblots and analyzed on reducing gels as mentioned previously (Banerjee et al., 2020b, 2021). Briefly, samples were denatured in a reducing sample buffer and analyzed on a reducing gel. Proteins were blotted from the gel onto polyvinylidene difluoride (PVDF) membranes (Immobilon, EMD Millipore) and detected using primary and secondary antibodies. Primary antibodies used were: 1:1000 mouse anti-SARS/SARS-CoV-2 N (ThermoFisher Scientific; Catalog number: MA5-29981; RRID: AB_2785780), 1:1000 rabbit anti-beta-actin (Abcam; Catalog number: ab8227; RRID: AB_2305186), and 2 μg/mL of mouse anti-ACE2 (R&D Systems; Catalog: MAB933; RRID: AB_2223153). Secondary antibodies used were: 1:5000 donkey anti-rabbit 800 (LI-COR Biosciences; Catalogue number: 926-32213; RRID: 621848) and 1:5000 goat anti-mouse 680 (LI-COR Biosciences; Catalogue number: 925-68070; RRID: AB_2651128). Blots were observed and imaged using Image Studio (LI-COR Biosciences) on the Odyssey CLx imaging system (LI-COR Biosciences).

#### Cell line and culture condition

The human lung adenocarcinoma cell line, Calu-3 (ATCC HTB-55) were grown in Minimum Essential Medium (MEM)-α (Gibco, Gaithersburg, Maryland) supplemented with 10% fetal bovine serum (FBS), and 1% penicillin-streptomycin (P/S) in 5% CO_2_ at 37°C. Media was replaced every 2 days and the cells were subcultured every 5 days. The cells were passaged and used in experimental assays without additional STR authentication or mycoplasma testing.

#### CRISPR targeting of *ACE2* regulatory elements *in vitro*

All sgRNAs flanking human *ACE2* regulatory elements and sub-elements containing TF binding sites were designed using the Genetic Perturbation Platform (GPP) sgRNA design tool from Broad Institute (https://portals.broadinstitute.org/gpp/public/), synthesized by Integrated DNA Technologies, Inc (Coralville, Iowa), and cloned into the PX458 vector following published protocols (Ran et al., 2013). The sequence of all sgRNAs along with their chromosomal locations (hg19) are listed in Table S2.

Guide RNAs (see Table S2), flanking the A*CE2* regulatory elements and sub elements containing TF binding sites, were first tested for the ability to induce efficient deletions of the enhancer elements/sub elements in cultured Calu-3 cells (N = 3 biological replicates per assay). Calu-3 cells were maintained in MEM α media supplied with 10% FBS (Gibco) and 1% Pen/Strep (0.025%) and seeded in a six-well plate for 1- day prior to transfection. After culturing at 37°C with 5% CO_2_, the cells were scanned for GFP fluorescence under GFP-microscope at 24 h to verify successful transfection efficiency (i.e., >70% of the cells were GFP positive). After 48 h of CRISPR experiment, DNA was extracted from the CRISPR-cas9 targeted Calu-3 cells using E.Z.N.A Tissue DNA Kit (Omega Bio-Tek, Norcross, GA), and the *ACE2* regulatory element/sub-element region was amplified using PCR primers flanking each sgRNA location (listed in Table S2). PCR amplified products were purified from 1% agarose gel using E.Z.N.A Gel Extraction Kit (Omega Bio-Tek, Norcross, GA). Sanger sequencing was used to verify successful deletion of the target region. To examine effects on *ACE2* and nearby gene expression (*TMEM-27* and *BMX1*), RNA was extracted from control and CRISPR-Cas9 targeted Calu-3 cells (N = 3 biological replicates, with 3 technical replicates per experiment per condition) and prepared using Trizol Reagent (Thermo Fisher Scientific, Springfield Township, New Jersey) and Direct-zol™ RNA Miniprep kit (ZYMO). Two micrograms of total RNA were used to synthesize first-strand cDNA using Super-Script III First-Strand Synthesis System (Thermo Fisher Scientific). qRT-PCR analysis was then performed with gene specific primers and Applied Biosystems Power SYBR master mix (Thermo Fisher Scientific) with *ACTB* house-keeping gene as an internal control. sgRNAs and primers used for qRT-PCR are listed in Table S2.

#### CRISPR deletion of *ACE2* regulatory elements under interferon treatment

Briefly, the Calu-3 cells were cultured in MEM α media supplemented with 10% FBS (Gibco) and 1% Pen/Strep (0.025%), plated in 6-well plates and utilized at ∼70% confluence. The cells were then subjected to CRISPR-Cas9 mediated deletion of *ACE2* regulatory elements/sub-elements in the presence/absence of recombinant protein of human interferon, alpha 2 (IFNA2) (OriGene Technologies Inc, Atlanta, GA) (100 ng/mL) for 48 h. Following CRISPR-deletion under interferon treatment, DNA and RNA were extracted from the Calu-3 cells and used for genotyping and gene expression analysis, respectively.

#### CRISPR deletion of *ACE2* regulatory elements under H_2_O_2_ treatment

The Calu-3 cells were cultured in MEM α media as described above and subjected to CRISPR-cas9 mediated deletion of *ACE2* regulatory elements/sub elements for 48 h. CRISPR-cas9 targeted Calu-3 cells were treated with hydrogen peroxide (H_2_O_2_) using the protocol described previously (Boardman et al., 2004). Following CRISPR deletion of *ACE2* regulatory elements/sub elements, the Calu-3 cells were challenged with or without oxidative stress by exposure to 0.5 mM (initial dose) of H_2_O_2_. As Calu-3 cells rapidly metabolize H_2_O_2_ in 1 h, H_2_O_2_ treatments were performed for 2 h, with additional bolus of H_2_O_2_ every 60 min for times longer than 1 h. DNA and RNA were extracted from the CRISPR-cas9 targeted Calu-3 cells subjected to H_2_O_2_ treatment, and used for genotyping and gene expression analysis, respectively.

### Quantification and statistical analysis

For CRISPR targeting experiments, all genes were quantified by real-time PCR using gene specific primers. *ACE2* gene expression data was normalized relative to *ACTB* house-keeping gene expression and compared between control and experimental condition (e.g., putative enhancer deletion or putative enhancer deletion in the presence/absence of IFN-α and H_2_O_2_ treatment). Normalization of *ACE2* gene expression and relative fold change calculation were performed by 2^−ΔΔCT^ method of Livak and Schmittgen (Livak and Schmittgen, 2001). All data are presented as the mean ± SEM, or in boxplot form indicating median, upper and lower quartiles. Individual pairwise comparisons between control and experimental condition were analyzed by two-sample, two-tailed Student’s T-test unless otherwise noted, with p < 0.05 regarded as significant. N numbers listed in figure legends (N = 9 biological replicates per comparison).

## Data Availability

• Accessions for publicly-available datasets used in this study are described in previous publications and in the key resources table. • This paper does not report original code. Code used to generate figures is available upon reasonable request from the lead contact. • Any additional information required to reanalyze the data reported in this paper is available from the lead contact upon request. • Additional Supplemental Items are available from Mendeley Data: https://data.mendeley.com/datasets/wmv6f24xm2/1. Richard, Daniel (2022) “Intronic regulation of SARS-CoV-2 receptor (ACE2) expression mediated by immune signaling and oxidative stress pathways - Richard et al., 2022”, Mendeley Data, V1: https://doi.org/10.17632/wmv6f24xm2.1.

## Supplemental information

Document S1. Figures S1–S5

Table S1. Microarray expression analyses, related to Figures 1 and 3 Sheet 1: Metadata for microarray expression datasets downloaded and processed for this study. Sheet 2: The top 200 genes co-expressed with *ACE2* expression (by Pearson correlation) across the indicated expression analysis (relevant main/supplemental figure also indicated). Sheet 3: enrichR gene-set enrichment results for gene sets shown in Sheet 2 – statistical results correspond with enrichments visualized in indicated main/supplemental figures.

Table S2. ACE2 regulation and experimental data, related to Figures 2 and 4 Sheet 1: CRISPR Guide and PCR primer information. Sheet 2: Primers used for real-time PCR. Sheet 3: Gene expression data for genes nearby *ACE2* following deletion of the indicated regulatory region. ‘VC’ – comparisons of deletion to vector-control samples (see STAR Methods). Sheet 4: Expression of full-length *ACE2*, and the dACE2 isoform, following either IFN treatment or H_2_O_2_ treatment – results correspond to Figures S3 and S5, respectively. Sheet 5: Expression of full-length *ACE2*, and the dACE2 isoform, following deletion of the indicated region, either in the presence or absence of IFN treatment. Sheet 6: Expression of full-length *ACE2*, and the dACE2 isoform, following deletion of the indicated region, either in the presence or absence of H_2_O_2_ treatment.

## Footnotes

Supplemental information can be found online at https://doi.org/10.1016/j.isci.2022.104614.
