# Supplementary material for: Intronic regulation of SARS-CoV-2 receptor (ACE2) expression mediated by immune signaling and oxidative stress pathways
Source: iScience. 2022 Jun 15;25(7):104614. doi: 10.1016/j.isci.2022.104614 (PMC9213013; doi:10.1016/j.isci.2022.104614)
Supplement: Document S1. Figures S1–S5 [file mmc1.pdf]

iScience, Volume *25*

Supplemental information

Intronic regulation of SARS-CoV-2 receptor (ACE2) expression mediated by immune signaling and oxidative stress pathways

Daniel Richard, Pushpanathan Muthuirulan, Jennifer Aguiar, Andrew C. Doxey, Arinjay Banerjee, Karen Mossman, Jeremy Hirota, and Terence D. Capellini

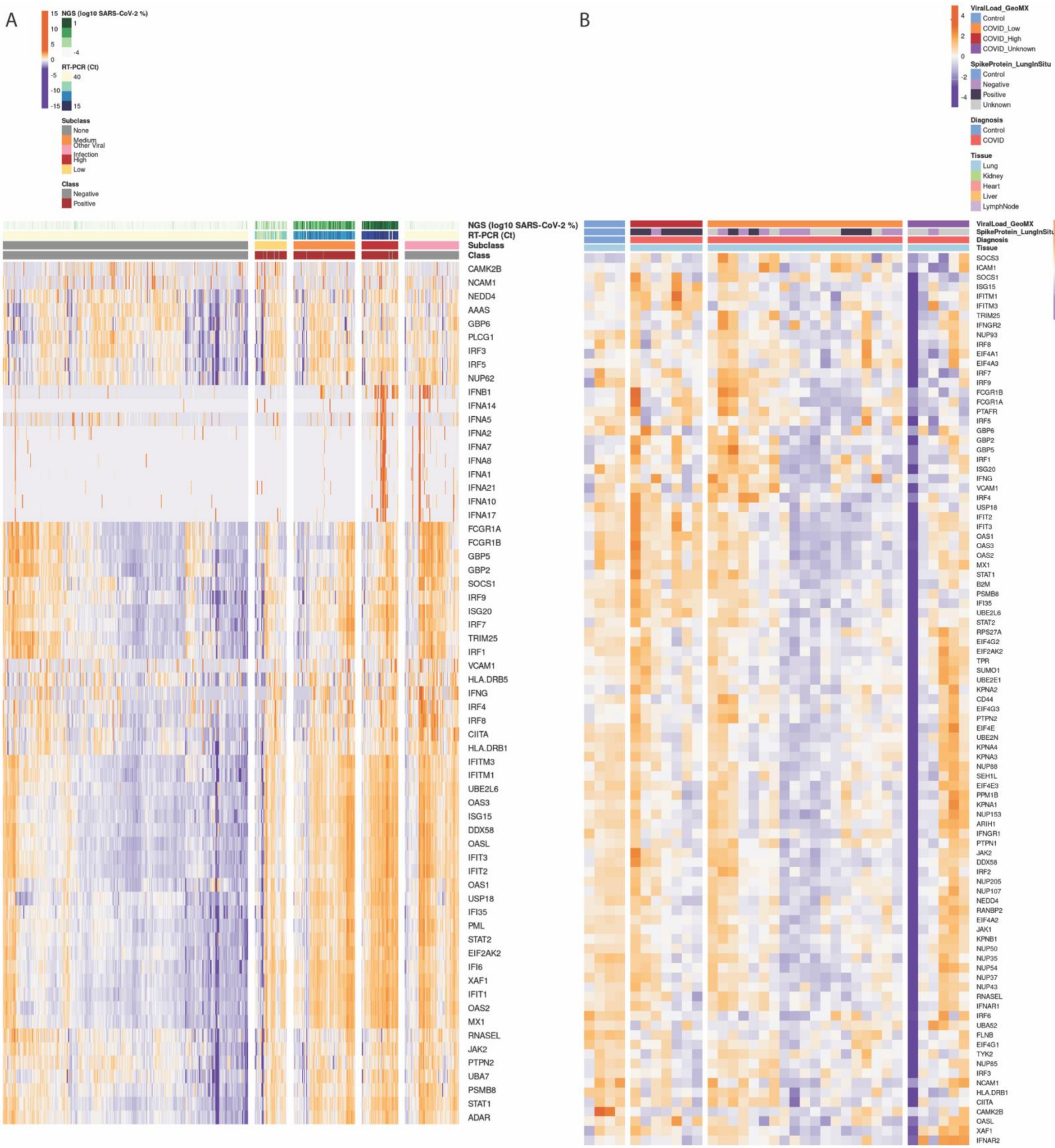

Figure S1. Interferon signaling in SARS-CoV-2 expression datasets. Related to Figure 1.

Expression heatmap of genes in the Reactome “Interferon signaling” term. Red/blue colour scale indicates normalized expression values (TPM). Generated via the ‘COVID19 Genes’ web interface by the Mason lab at Cornell University (cit). (A) Nasopharyngeal (NP) swab data. (B) Lung tissue autopsy data.

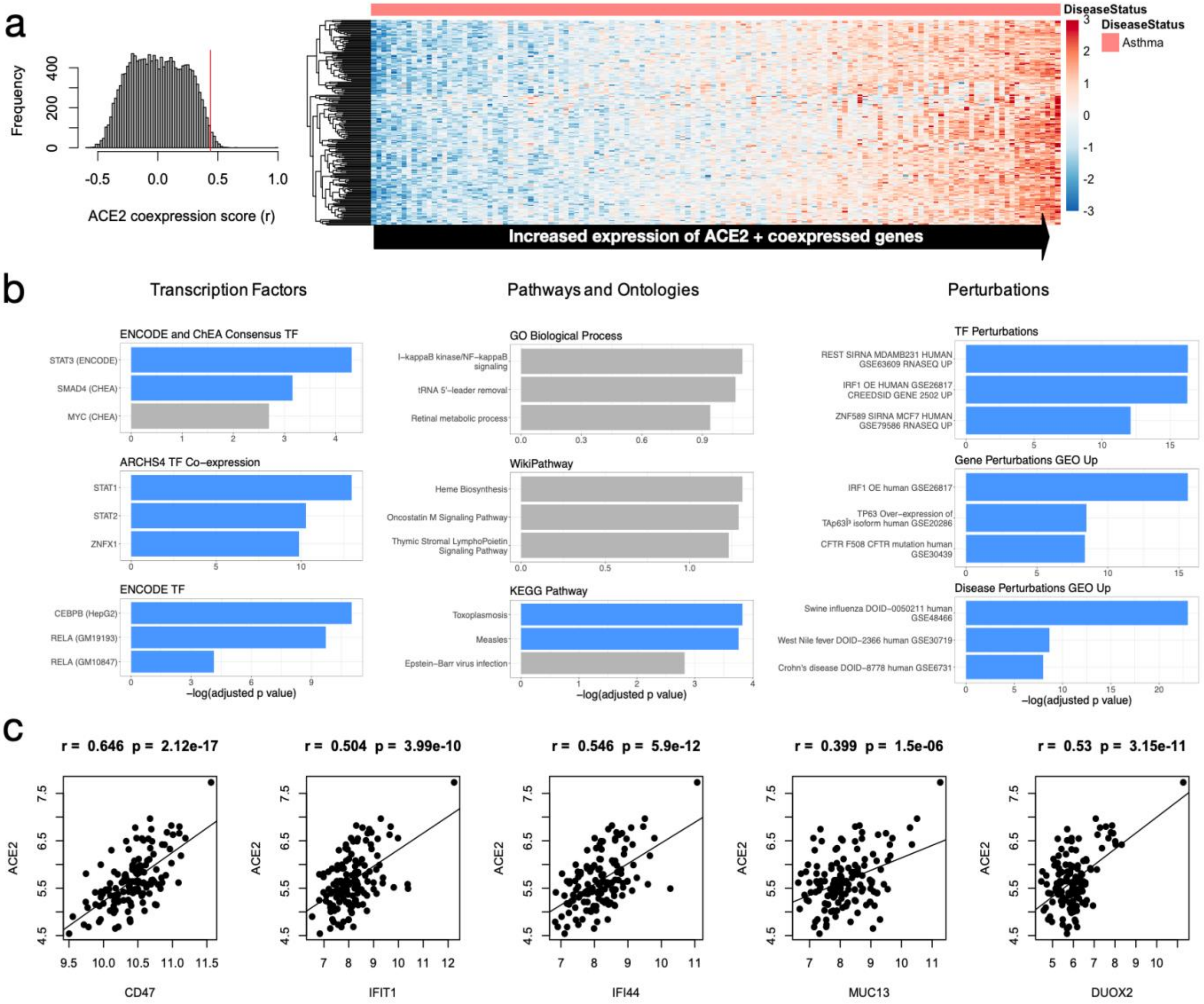

Figure S2. *ACE2* co-expressed genes and functional associations in asthmatics. Related to Figure 1.

a) Expression of top 200 *ACE2*-correlated genes (including *ACE2*) in asthmatics (N=136). **b)** Functional enrichment analysis of top 200 *ACE2*-correlated genes (including *ACE2*). Terms are ranked by -$\log_2$(FDR-adjusted *p* value) for nine ontologies/groups of interest. **c)** Pearson correlation of *ACE2* with important interferon-related candidate genes found to be co-expressed with *ACE2.*

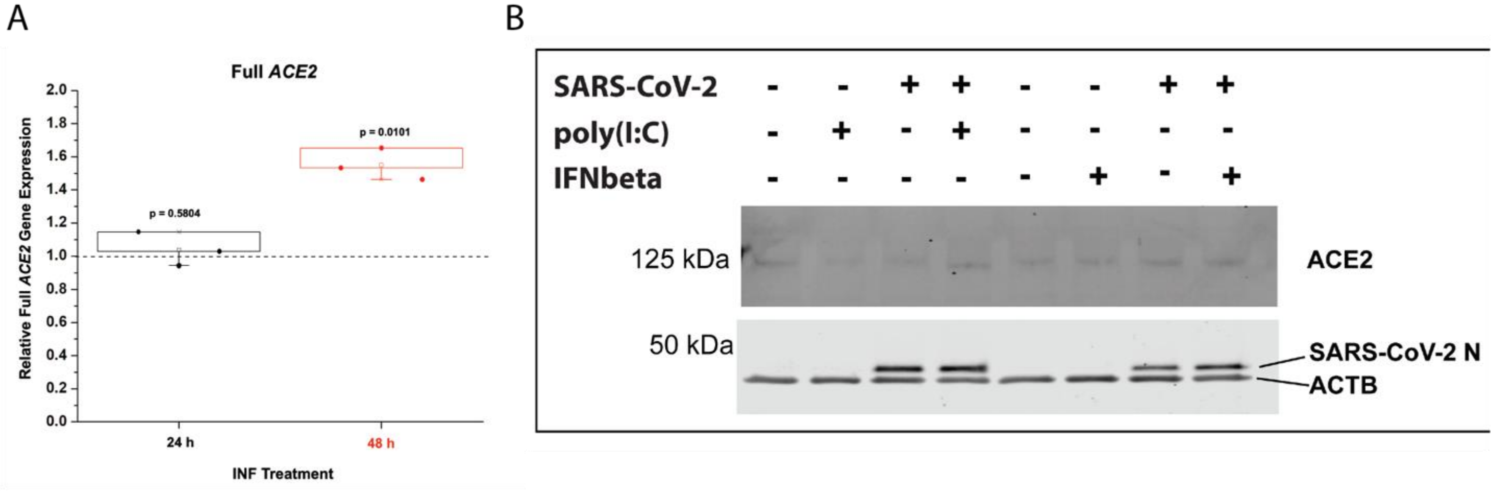

Figure S3. ***ACE2* expression in response to immune signaling**. Related to Figure 2. (**A**) Expression of full-length *ACE2* following 24 or 48h of IFN-α treatment – p-values of expression change relative to untreated controls. P-values indicate results of two-tailed Student's t-test. (**B**) Immunoblots for ACE2 protein in Calu3 cell lysate in the context of SARS-CoV-2 infection (12h culture), poly(I:C) treatment (6h), and/or IFNβ1 treatment (6h). See STAR Methods.

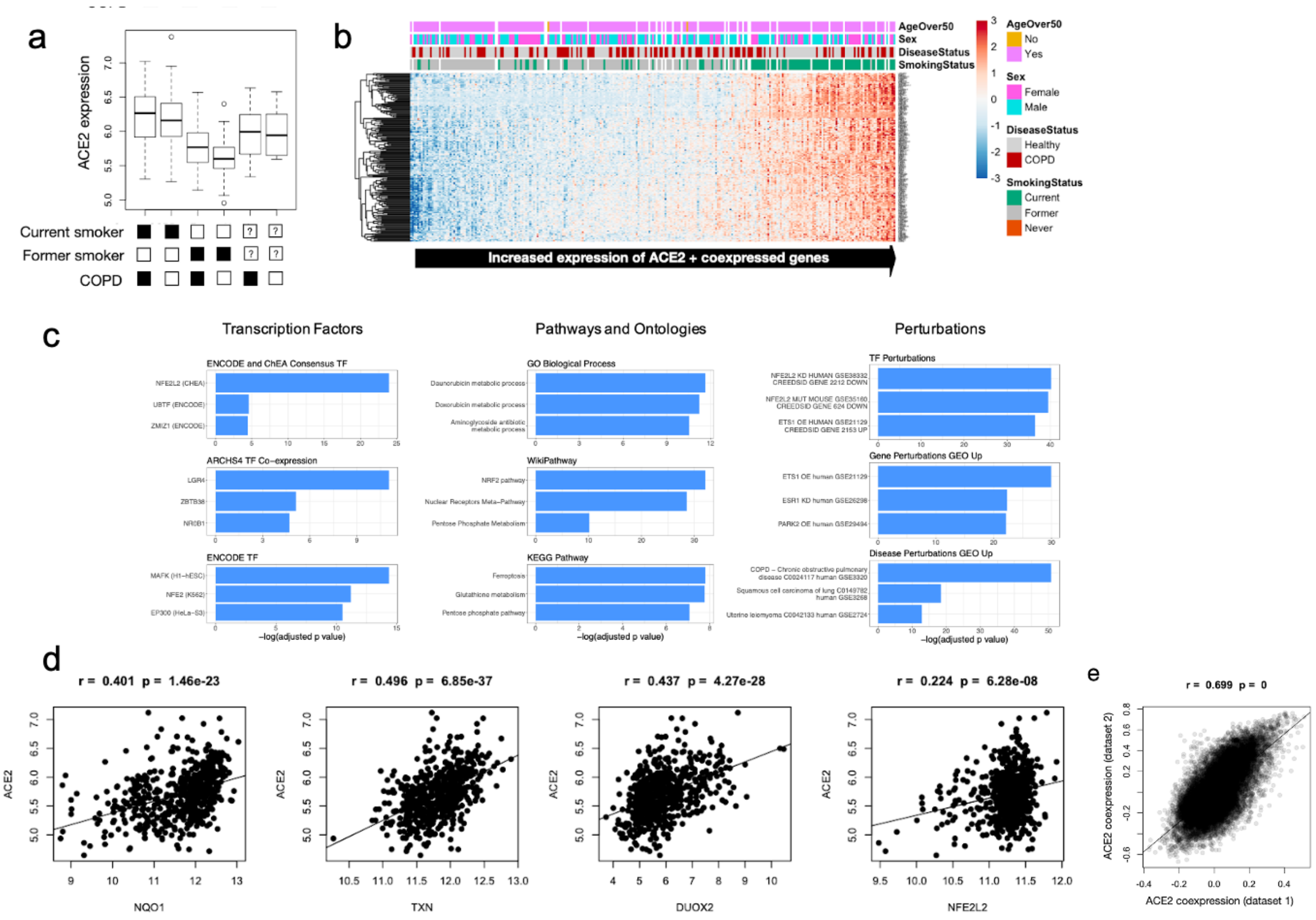

Figure S4. Expression and functional enrichment analysis of *ACE2* and co-expressed genes in smokers and individuals with COPD. Related to Figure 3. a) Analysis of relative *ACE2* expression with respect to smoking status and COPD diagnosis. **b)** Expression of top 200 *ACE2*-correlated genes (including *ACE2*) individuals with various smoking status and COPD diagnosis (N=345). **c)** Functional enrichment analysis of top 200 *ACE2*-correlated genes (including *ACE2*). Terms are ranked by -$\log_2$(FDR-adjusted *p* value) for nine ontologies/groups of interest. **d)** Pearson correlation of *ACE2* with important interferon-related candidate genes found to be co-expressed with *ACE2.* **e)** Correlation between dataset 1 (N=159, Figure 3) and dataset 2 (N=345, Figure S3).

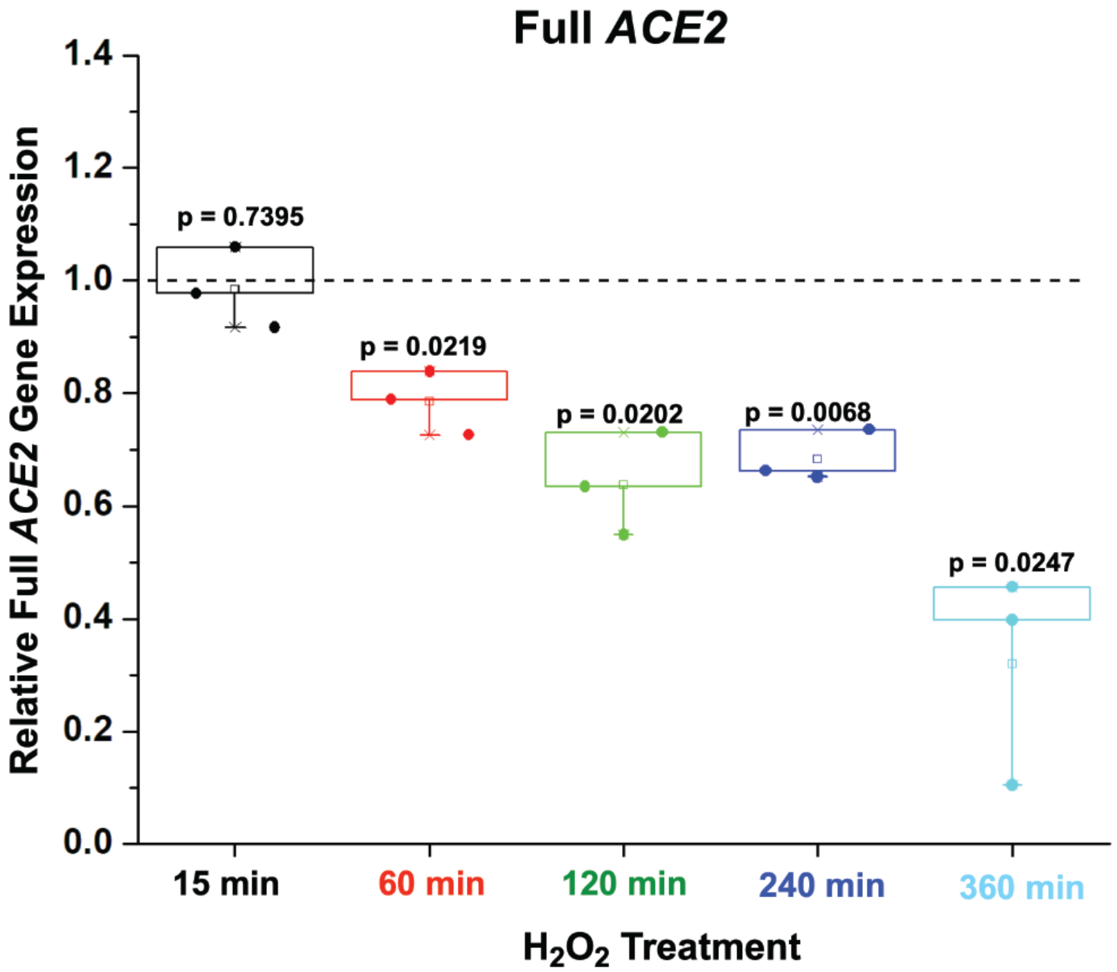

Figure S5. *ACE2* expression in response to oxidative stress. Related to Figure 3. Expression of *ACE2* in Calu-3 cells (relative to untreated controls) following H_2O_2 treatment for the indicated length of time (see Methods, Table S2). P-values indicate results of two-tailed Student's T-test.
